# Postanoxia-Induced Chorea Treated with Intravenous Fentanyl

**DOI:** 10.1155/2023/7652013

**Published:** 2023-04-11

**Authors:** Ashley Audi, Brittany Cunningham, Christopher Newey

**Affiliations:** ^1^Section of Medicine, Northeast Ohio Medical University, Rootstown, Ohio, USA; ^2^Section of Pharmacy, Cleveland Clinic Akron General Hospital, Akron, Ohio, USA; ^3^Department Chair of Neurocritical Care and ICU-EEG, Sanford USD Medical Center, Sioux Falls, South Dakota, USA

## Abstract

The case presented is that of a young male with postanoxic brain injury secondary to cocaine overdose who began to exhibit choreiform movements of the left upper extremity. Traditional treatment options for chorea were unsuccessful, leading to the administration of fentanyl, which rapidly resolved the patient's choreiform movements. There is a limited research involving the treatment of chorea in anoxic brain injury as well as fentanyl's role in the movement pathway. We hypothesize that chorea can be caused or exacerbated by opioid withdrawal in a patient with chronic opioid use through modulation of dopamine transmission.

## 1. Introduction

**Figure 1:**
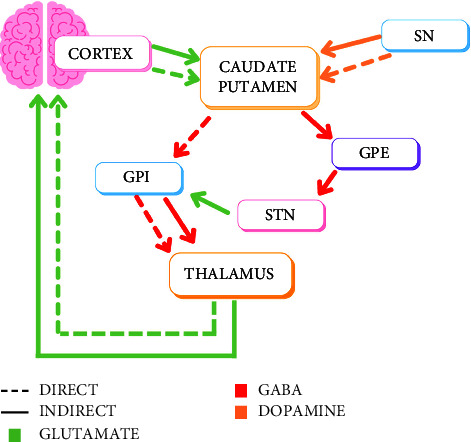
Direct and indirect pathways of the basal ganglia. The direct pathway, represented by the dashed line, begins in the cortex, sending a stimulatory signal to the caudate and putamen. The caudate and putamen then send inhibitory signals to the GPi which inhibit the thalamus, therefore inhibiting an inhibitor, leading to overall stimulation of movement. The indirect pathway, represented by the solid line, also begins by the cortex stimulating the caudate and putamen. The caudate and putamen then send an inhibitory signal to the GPe. The GPe inhibits the subthalamic nucleus which stimulates the GPi. Therefore, the end result of the indirect pathway is stimulation of the inhibitory GPi, leading to inhibition of movement. The substantia nigra acts on both pathways using dopamine, using D1 receptors to stimulate the direct pathway and D2 receptors to inhibit the indirect pathway. (SN, substantia nigra; GPE, globus pallidus externus; GPI, globus pallidus internus; STN, subthalamic nucleus).

The basal ganglia serve a key role in the movement pathway. These pathways are classically shown as the indirect and direct pathways (Figure 1). In general, the direct pathway stimulates movement while the indirect pathway inhibits movement. Damage to any of these nuclei will create a movement disorder. One such movement disorder called chorea can result from damage to either the caudate, subthalamus, and/or globus pallidus.

Chorea is a movement disorder characterized by involuntary, excessive, spontaneous movements that are abrupt and randomly distributed with irregular timing. The word shares a common Latin origin as choreography, describing movements that appear similar to dancing [1]. Chorea occurs in a wide array of disease processes. It can be due to neurodegenerative disease, drugs such as levodopa, anticonvulsants, antipsychotics, cocaine, and amphetamines or toxins such as carbon monoxide and alcohol intoxication. Additionally, it can be secondary to rheumatic fever, infection, metabolic and endocrine abnormalities, or ischemia [1]. Chorea is also a rare complication of anoxic brain injury affecting the basal ganglia. The globus pallidus and putamen are areas of elevated metabolic demand with high concentrations of neurotransmitters which utilize large amounts of oxygen and glucose. Due to this, these areas are susceptible to hypoxic injury. In severe anoxic injury, damage extends beyond watershed zones to include the cerebral cortex, bilateral basal ganglia, and hippocampi [2].

Drugs of abuse, such as cocaine, can also affect neurotransmission by impairing the inhibitory GABA transmission in the movement pathway and increasing glutamate and dopamine transmission, inhibiting reuptake, and therefore increasing movement two-fold. Acute transient chorea in cocaine intoxication is described as self-limited choreoathetoid movements of the face and limbs which can last days. Patients with cocaine overdoses have been demonstrated to have increased glutamate peaks, showing intense excitotoxicity in bilateral pallidi [3, 4]. Here we present a young patient with chorea following an anoxic brain injury due to a cocaine overdose that resolved following the administration of fentanyl. His magnetic resonance imaging (MRI) shows restricted diffusion in multiple areas including the globus pallidus bilaterally. We suspect that to be the etiology of the chorea.

## 2. Case Report

This is a 27-year-old male with a past medical history of cocaine and fentanyl abuse who presented to the ED after a suspected drug overdose. The patient was found down in the field, unconscious with a last known well of 6:30 pm the prior evening. It was unclear how long the patient had been unconscious for as he lives alone. In the field, he was given naloxone with minimal improvement; he received 8 mg of naloxone total and was intubated for airway protection. On presentation, his blood pressure was found to be 176/94 mm Hg, heart rate of 144 BPM, respiratory rate of 22 BPM, and O_2_ saturation of 73%. Labs revealed a CK 2454 U/L, WBC 32.03 k/*μ*L, and venous blood gas showing a pH of 7.14 with a CO_2_ 69.4. Urine toxicology was positive for cocaine and cannabis. His initial CT revealed bilateral occipital hypodensities and was concerning for possible posterior reversible encephalopathy syndrome (PRES). He was admitted to the medical intensive care unit (MICU) and treated for rhabdomyolysis and acidosis.

**Figure 2:**
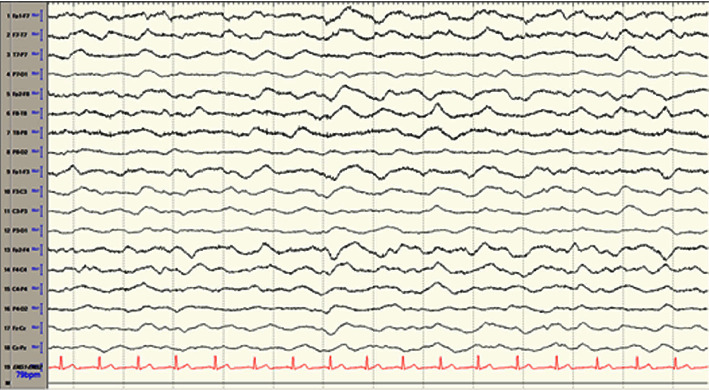
Continuous electroencephalography (CEEG) showing a continuous delta slowing maximum in the bifrontal regions captured before the onset of choreiform movements. The patient was not on any continuous sedation during this period that would alter their level of consciousness. No seizure activity noted. (Bipolar montage; LF 1.6 Hz, HF 70 Hz, sensitivity 7 uV; 15 s Epoch).

**Figure 3:**
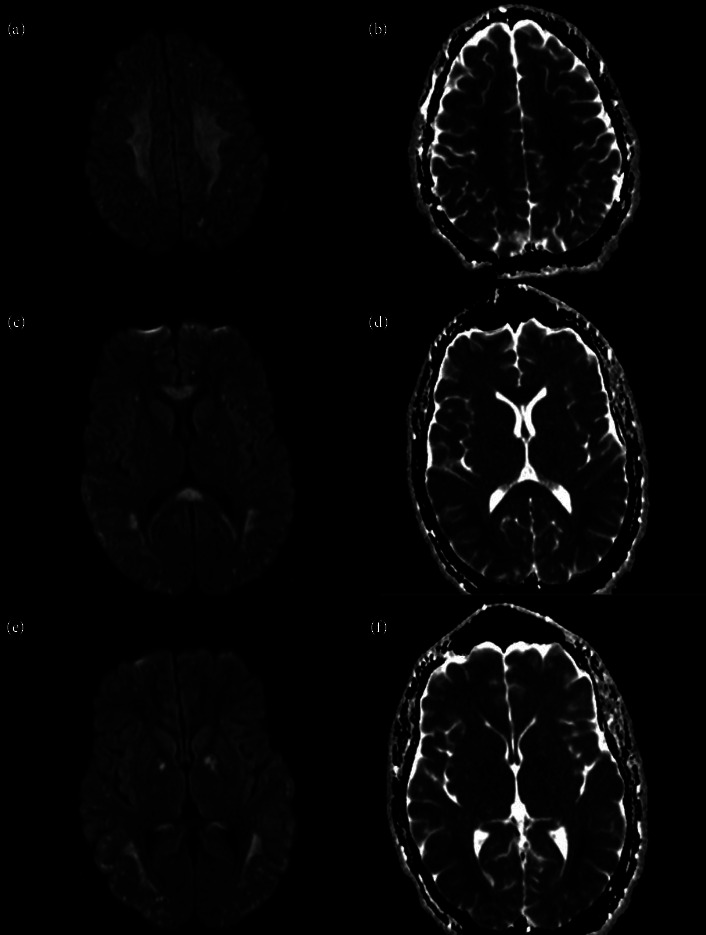
Magnetic resonance imaging (MRI) of the brain showing diffusion weighted imaging (DWI; images (a), (c), and (e)) and apparent diffusion coefficient (ADC; images (b), (d), and (f)) sequences. Restricted diffusion noted in the subcortical white matter (a and b), corpus callosum (c and d), and globus pallidus (e and f).

In the MICU, EEG revealed bilateral cortical dysfunction, max in the left frontotemporal region and severe diffuse encephalopathy with no epileptiform discharges (Figure 2). The patient's echocardiogram was normal. During his stay, the patient remained encephalopathic and experienced complications including pneumonia and acute kidney injury (AKI). He remained unable to follow commands. An MRI completed on hospital day 5 revealed bilateral restricted diffusion in the cerebral white matter, and bilateral globus pallidus, corpus callosum, and bilateral hippocampus in the medial temporal lobes with scattered punctate restricted diffusion in the bilateral cortex and cerebellum consistent with posthypoxic leukoencephalopathy and global hypoxic ischemic injury (Figure 3). On repeat examination in the following days, the patient developed severe choreiform movements of the left upper extremity as well as episodes of fever, tachypnea, tachycardia, hypertension, and bladder spasms consistent with paroxysmal sympathetic hyperactivity following traumatic brain injury.

### 2.1. Treatment Plan and Outcomes

Initial medical management consisted of dexmedetomidine and aripiprazole for movement suppression. Despite this, choreiform movements and episodes of sympathetic hyperactivity continued. To further manage these symptoms, a fentanyl infusion was started at 150 mcg/hr. Surprisingly, following the infusion, his choreiform movements resolved. While on fentanyl, the patient would still open eyes to verbal stimuli, however, no meaningful activity was observed. Chorea returned when the infusion was paused for spontaneous breathing trials. Toluene levels and an extended drug panel were sent with no significant findings. He ultimately had a tracheostomy and percutaneous endoscopic gastrostomy (PEG) placed and was transferred to a long-term acute care facility. The patient had a poor prognosis, failed repeated spontaneous breathing trials, and was unable to follow commands. He did not attend or track when you entered the room and did not appear to have any meaningful movements; his brainstem reflexes were intact. Fentanyl served multiple purposes in his case for pain control as well as treatment of the paroxysmal sympathetic hyperactivity. Fentanyl was ultimately weaned using oral opiates, and the patient was discharged to a long-term acute care hospital with aripiprazole and oxycodone.

## 3. Discussion

There is limited data on the treatment of chorea in anoxic brain injury. Available literature focuses on the treatment of chorea associated with Huntington's disease and has discussed medication management of chorea primarily through manipulation of dopamine transmission. In the United States, tetrabenazine/deutetrabenazine are the only current FDA-approved medications for chorea treatment and work by inhibiting vesicular monoamine transporter 2 (VMAT2), decreasing dopamine release at the presynaptic membrane. Targeting other areas of the movement pathway have been studied and tried in clinical practice. Atypical antipsychotics work primarily by blocking the D2 receptors, inhibiting the direct pathway of movement. Atypical antipsychotics are the first choice internationally despite their off-label use [5]. In this case of anoxic injury, aripiprazole was trialed without improvement in symptoms. We ultimately found that fentanyl improved our patient's chorea.

Endogenous opioid peptides (EOP) have been suggested to be involved in the motor pathway, with degeneration of EOP-containing neurons in disorders such as Parkinson's disease and Huntington's chorea and excessive opioid activity in movement disorders such as tardive dyskinesia. The basal ganglia contain high concentrations of opioid receptors and enkephalins [6]. In animal studies, it has been demonstrated that by destroying nigrostriatal tracts, opioid receptors are reduced by 30% without loss of enkephalins. This suggests that these opioid receptors may be on presynaptic dopamine nerve terminals whereas enkephalins lie within the striatum. These opioid receptors, therefore, may have the ability to modulate dopamine synthesis and release [7]. In addition to opioid receptor location on presynaptic membranes, they have also been found on dopamine-containing neuron cell bodies. By administering opioids to rats, animals showed signs of decreased dopamine transmission such as akinesia [6].

It is traditionally understood that in the setting of increased dopamine, the direct pathway of movement is stimulated, and the indirect pathway is inhibited, leading to stimulation of movement. However, a case report has demonstrated that discontinuation of chronic opioid use can lead to a hyperkinetic state that rapidly resolves with the administration of IV fentanyl. It was hypothesized that this may be due to opiate deficiency, insensitivity of the opiate receptors, and upregulation of receptors secondary to tachyphylaxis, leading to hyperkinesia [8]. Our patient was known to have a chronic history of fentanyl use; however, urinary drug screening in the emergency room did not reveal evidence of acute opioid intoxication. It is possible that due to the rapid discontinuation of fentanyl in our patient, he developed chorea through the above mechanism. In addition to the potential benefits in the movement pathway, fentanyl is also used in the management of paroxysmal sympathetic hyperactivity in postanoxic brain injury, making this drug a reasonable choice for our patient [9].

In this case, the patient had failed traditional therapy for chorea with the initiation of the dopamine antagonist aripiprazole. He also continued to experience symptoms with the initiation of dexmedetomidine. The chorea did resolve with administration of IV fentanyl. Given the half-life of aripiprazole is 75 hours. It will take approximately two weeks to get to steady state. In our case, IV fentanyl was used as an abortive agent to acutely control chorea and was ultimately able to be weaned to oral opiates and aripiprazole on discharge [10].

## 4. Conclusion

Our case highlights the unique finding of chorea following anoxic brain injury due to drug overdose. Typical medications used to treat chorea were not successful and the patient developed medical complications of pneumonia, AKI, and paroxysmal sympathetic hyperactivity and was unable to be successfully extubated. Ultimately, trialing fentanyl improved the choreiform movements quickly and helped treat his concomitant conditions.
